# Exploring the Challenges and Opportunities of Adopting and Using Telemedicine for Diabetes Care and Management: Qualitative Semistructured Interview Study Among Health Care Providers and Patients With Diabetes

**DOI:** 10.2196/46324

**Published:** 2023-09-07

**Authors:** Rabab Altabtabaei, Dari Alhuwail

**Affiliations:** 1 Information Science Department Kuwait University Kuwait City Kuwait; 2 Health Informatics Unit Dasman Diabetes Institute Kuwait City Kuwait

**Keywords:** health, telemedicine, diabetes, challenges, Kuwait, technology, informatics, diabetes care, management, obstacle, health care provider, qualitative study, mobile phone

## Abstract

**Background:**

Around the world, over half of the global population experiences noncommunicable diseases, resulting in premature death. Health care providers (HCPs) can deliver medical treatment from a distance by using digital advancements such as telemedicine. However, there is a limited understanding of the difficulties and opportunities of implementing telemedicine solutions in different socioeconomic and cultural environments, including Kuwait.

**Objective:**

The purpose of this study is to (1) examine the obstacles and benefits of telemedicine in the context of diabetes treatment and management, as perceived by both HCPs and patients with diabetes; (2) investigate the nonfunctional requirements for telemedicine applications used in diabetes care and management; and (3) provide suggestions to enhance the integration and adoption of telemedicine in Kuwait’s health care system for diabetes care and management.

**Methods:**

The research used a qualitative and exploratory design, with semistructured interviews as the main data collection method. Participants were recruited on the internet through social media platforms due to the COVID-19 pandemic. The results were analyzed using thematic analysis and the Framework Method. The “diffusion of innovation” model was used as a perspective to interpret the findings.

**Results:**

A total of 20 participants were included in this study—10 HCPs and 10 patients with diabetes—all of whom supported telemedicine. The HCPs reported that many diabetes cases could be managed through telemedicine, with only a few requiring in-person visits. Patients with diabetes noted the convenience and time-saving aspect of telemedicine. Both groups recommended the creation of a secure and user-friendly telemedicine system similar to popular social media platforms. Additionally, participants emphasized the importance of telemedicine during the pandemic as a way to prioritize patient safety.

**Conclusions:**

The results of this study provide valuable insights into the needs and preferences of both HCPs and patients with diabetes in a resource-rich country like Kuwait to embrace telemedicine. The COVID-19 pandemic has changed the way medical care is provided and has pushed both groups to consider digital solutions for ongoing diabetes management and treatment.

## Introduction

Globally, more than one-half of the world's population experience noncommunicable diseases (NCDs) causing premature death [1,2]. Diabetes, cancer, cardiovascular diseases, and chronic respiratory diseases are considered the main types of NCDs [3]. The International Diabetes Federation estimates that about 463 million individuals worldwide experience diabetes, with this number expected to reach 700 million individuals by 2045 with 681,100 reported cases in the State of Kuwait [4,5]. This results in an economic burden for individuals with diabetes as each would spend around US $2000 for treatments [6]. According to the World Health Organization [7], diabetes is solely responsible for 1.5 million deaths in 2019; almost half of all deaths due to diabetes happen before the age of 70 years.

Self-management and lifestyle adjustments are essential for patients with NCDs [8,9]; regular checkups with physicians are mandatory to ensure health status. However, regular checkups are time and effort-consuming and may be inconvenient for patients to attend [10]. With the technology available today, physicians can provide medical care remotely to patients and help save resources through telemedicine [11]. The World Health Organization [12] defines telemedicine as “the delivery of health care services, where distance is a critical factor, by all health care professionals using information and communication technologies for the exchange of valid information for diagnosis, treatment, and prevention of disease and injuries, research and evaluation, and for the continuing education of health care providers (HCPs), all in the interests of advancing the health of individuals and their communities.”

As technology is rapidly evolving, telemedicine consultations are now carried out via video calls from laptops or smartphones [13]. It promises patients to deliver remote medical care over long distances at less cost [14]. For NCDs management, telemedicine solutions can improve self-management, increase patient satisfaction with receiving medical test results from home, enhance diabetes control, and reduce stress [9,15-17]. During the COVID-19 pandemic, telemedicine was reintroduced into the health care system globally and intensively. Kuwait was one of the countries that had not systematically introduced telemedicine in hospitals until the early start of the pandemic, when a large portion of HCPs provided health care services using telemedicine technology as a comfortable and safe alternative to physical visits [18-21].

While studies have proven the efficiency of using telemedicine, several barriers are limiting the adoption of this technology [16]. Costs, technical issues (eg, internet speed and poor user experience), concerns for privacy and security, and patient population (eg, older patients and those that face difficulties using technology) were described as major barriers [22,23]. Especially for rural communities, internet inaccessibility can be a major barrier [24]. Lifestyle, cultural beliefs, values, and social factors are crucial in managing diabetes and should be considered carefully for any intervention to be successful [25,26]. While opportunities exist for leveraging telemedicine for diabetes care and management, there is still little known about the contextual challenges and opportunities of leveraging telemedicine solutions in varying socioeconomic and cultural contexts, including the State of Kuwait.

This research aims to (1) examine the obstacles and benefits of telemedicine in the context of diabetes treatment and management, as perceived by both HCPs and patients with diabetes; (2) investigate the nonfunctional requirements for telemedicine applications used in diabetes care, and management; and (3) provide suggestions to enhance the integration and adoption of telemedicine in Kuwait’s health care system for diabetes care and management.

## Methods

### Study Design

This study used a case study approach [27] to answer questions related to “how” and “why” telemedicine is worth the experience for patients with diabetes from the perspectives of patients and HCPs. Through semistructured interviews, this study primarily used a qualitative and exploratory design to uncover rich context-specific findings. This study followed the social science theory of “diffusion of innovation” [28] which is highly used in measuring the population’s acceptance of adopting a new system or innovation [29]. Driven by the “diffusion of innovation” theory, the questions were derived and inspired by previously published studies [30-32].

### Data Collection

This study followed a purposeful convenience sampling approach that considered a random selection of participants with diverse backgrounds, roles, and demographics. Interviews were carried out from December 2020 to July 2021 during the COVID-19 pandemic. Using Instagram and WhatsApp, the researchers recruited the participants; contacts were made with participants either via direct messages or email. Based on the participants’ preferences, interviews have been conducted either face-to-face, over the phone, or on the internet using Microsoft Teams or Zoom apps (Zoom Video Communications, Qumu Corporation).

Before conducting an interview, each participant signed a consent form explaining the purpose of this study and how the information obtained will be protected. In cases where obtaining the participant’s signature was not possible (phone or on the internet), the participant was presented with the consent form and the interview commenced only after the participant agreed. Each interview involved 1 participant and lasted for approximately 35 (SD 11.75) minutes on average. The interviews were conducted in English or Arabic based on the participant’s preference, except for HCPs in English. Both face-to-face and phone interviews were audio-recorded and transcribed verbatim for analysis purposes.

The interviews with HCPs started with general technology used in the workplace before the pandemic. On the other hand, the interviews with patients with diabetes started with general diabetes management questions. The questions that followed the introductory questions were related to the participant’s knowledge about telemedicine’s challenges, nonfunctional requirements, challenges during the pandemic, and their suggested recommendations for improving telemedicine adoption and uptake; the questionnaires can be found in Multimedia Appendices 1 and 2.

### Data Analysis

The analysis of the transcripts followed a thematic analysis approach and used the Framework Method [33,34]. This approach allowed the researchers to incorporate the perspectives and viewpoints of a diverse group of participants systematically. Initially, the analysis involved the familiarization of the researchers, independently, with the interview transcripts searching for basic observations and patterns in the data, and coding them accordingly. Iteratively, the researchers reviewed the coding concepts and grouped them into themes. The researchers met regularly to discuss, compare, corroborate, and revise codes and themes. Saturation was achieved at approximately the seventh and eighth interviews for patients with diabetes and HCPs, respectively. However, we preferred a cautious approach and continued the remaining interviews as planned.

### Ethics Approval

The ethics committee of the Ministry of Health approved this study (REC 2019/1187).

## Results

### Overview

In the following sections, we present the findings from the perspectives of HCPs and patients with diabetes separately. Overall, from December 2020 to July 2021, a total of 10 HCPs and 10 patients with diabetes agreed to participate in this study. Refer to Table 1 for a summary of the HCPs’ demographics and Table 2 for a summary of the patients’ demographics.

**Table 1:** Demographics of health care providers participants.

Health care providers (N=10)		Female (n=7), n	Male (n=3), n
**Age group (years)**			
	25-34	3	1
	35-44	2	2
	45-54	1	0
	55-64	1	0
**Role**			
	Dietitian	1	1
	Specialist	2	2
	Nurse	2	0
	Educator	2	0
**Years of experience**			
	1-10	4	1
	11-20	2	2
	>21	1	0

**Table 2:** Demographics of patients with diabetes.

Patients (N=10)		Female (n=5), n	Male (n=5), n
**Age group (years)**			
	25-34	0	3
	35-44	0	1
	45-54	2	0
	55-64	1	1
	65-74	1	0
	60-64	1	0
**Diabetes type**			
	Type 1	1	4
	Type 2	1	1
	Gestational	3	0
**Education level**			
	High school degree	3	0
	Diploma	2	2
	Bachelor	0	3

### Perspectives of HCPs

#### Challenges and Opportunities

##### Compatibility

In total, 9 participants agree that this technology is compatible in Kuwait (Q1*—*refer to Textbox 1 for selected representative quotes). In addition, 1 participant felt that foot diabetes may be difficult to apply (Q2).

**Representative quotes from participants. Q refers to quote; P refers to health care provider; C refers to patient with diabetes.**

**Verbatim quote**s:

- Q1: *I think it will improve patient care and probably improve the flow of clinics making them more flexible, I think I could see it being able to see a potentially larger volume of patients through virtual means rather than just because it does not increase efficiency*. [P6]
- Q2: *With a diabetic foot, we cannot tell anything because this patient today we need to see the wound because every day it is different, patient blood sugar also affects, some patients one day have stable sugar, somedays not, we cannot diagnose this telemedicine, diabetes it is possible but diabetic foot no*. [P10]
- Q3: *The patient will have fast contact with the doctor instead of waiting and going to the parking and booking appointment and waiting his turn*. [P8]
- Q4: *I do a lot of virtual care I have clinic on Tuesdays where we do a lot of telephone calls and I have to say that clinic I finish really quickly*. [P6]
- Q5: *Maybe if we had WhatsApp, we could make the visit short and give him instructions and advice I can tell him to come to emergency if they are feeling unwell*. [P9]
- Q6: *Infrastructure within the government whether this is a hospital-based or Ministry and the technology*. [P3]
- Q7: *The problem is in the implementation so if it is implemented right then it will be great but the problem with IT in our organization is their implementations are very poor*. [P7]
- Q8: *If we have to print the papers and then have to come to collect, then there is no benefit in doing virtual clinic*. [P7]
- Q9: *We also need people who are aware of technology we need classes to teach them how to use technology*. [P8]
- Q10: *The internet is very poor, so we find sometimes technical issues, the internet is off connection*. [P2]
- Q11: *[System name masked] is available in [hospital name masked] and [hospital name masked], [system name masked] system is bad it is not integrated with the labs or pharmacy just for the doctor to write in the file, for medication he needs to print paper and give to the patients. Also, there is no integration between hospitals*. [P4]
- Q12: *There shouldn't be any risks if it's used as a supporting method to help patients manage their care*. [P3]
- Q13: *Patients’ misunderstandings that lead to medical errors may be because it is new way of providing care so there may be more errors because the doctors and the patients are not used to it, but I think this is just a difficult in the beginning if we are cautious then that should not be a problem another thing of course if the implementation as I said*. [P7]
- Q14: *Unfortunately, my colleague hates it, a lot of them are camera shy and they don't like to appear in front of the camera they fear that maybe patients are recording the conversation behind the camera while you can't see them or they're taking pictures of them and spreading it over social media*. [P1]
- Q15: *My colleagues are already adopting this technology and we are all interested because this is where diabetes is going, in a conference they were talking about new technologies and how we adapted in diabetic education, diabetic clinic, at a visit, in follow-ups we are all into this approach*. [P4]
- Q16: *Safety is number one especially if I am going to use the system as also a payment portal for regarding to provide my services in the private sector*. [P5]
- Q17: *Now all the hospitals have their own system it is also safety that not everyone can view their record, it is safe that each hospital and each doctor can view only their patients record it is safety for the patient, but if the patient wants to go to another hospital, he can take permission from the doctor to take his record and he can go anywhere*. [P8]
- Q18: *If the system is implemented correctly people will know how to use it because people know how to use WhatsApp and Instagram and Twitter and all of these apps even older patients, they know how to use that because they are user-friendly*. [P7]
- Q19: *Accessibility of the system, uptime, being able to log in and everything working, the latency*. [P7]
- Q20: *I think we are using it now because it is probably safer than the patient coming in and getting covid, we probably need more data for specific patients like diabetes safety and what are the red flags that would need you to bring the patient in clinic*. [P6]
- Q21: *I think the biggest problem in safety is patients mix ups because a lot of patients have similar names, and this is an unrecognized problem in our hospitals in Kuwait a lot of patients have similar names ... we open the chart we start talking to the patients and prescribe medications and realize it is all in the wrong patient that can be huge problem*. [P7]
- Q22: *This is our big concern, for such cases the caregiver, we depend on the son, nanny or the nurse we contact them*. [P2]
- Q23: *We give them an option of the whether it is through Zoom or it is through the phone and most likely they pick that they want their consultation through the phone*. [P1]
- Q24: *Old people like to feel or see things not just speak they tend to be more emotional; I would rather have it as a video call maybe rather than just an audio or voice call*. [P5]
- Q25: *It has to be incorporated with the electronic medical records not as a separate way on how to deal with patients*. [P3]
- Q26: *Don’t make a lot of clicks, the easier it is the simple it is the better it is for the patients*. [P4]
- Q27: *Having good visuals either color relaxing for the eyes or easy on the eye*. [P5]
- Q28: *Make it simple and easy, easy to give information to the patients*. [P9]
- Q29: *Maybe alarming with blood glucose...a reminder would be good, especially now we are back to normal and if we want to continue with telemedicine*. [P2]
- Q30: *We need sign language for those deaf patients the doctor attends the appointment, but he does not have translator … and maybe the things to be heard for those who do not see*. [P7]
- Q31: *A lot of them were sleeping, not answering, not saying the right answers, why is your blood sugar high? Oh, what? I do not know… it's a vacation or whatever they say, the patient was not aware he does not have the awareness of stabilizing the blood sugar whether it is before or during COVID. So COVID did increase this problem under pretext of we are in pandemic and staying at home, so we overeat*. [P4]
- Q32: *The patient felt feared that no one will see them, or they don’t have the medications, but the good thing is we introduced telemedicine fast, this is number one, fast introduction*. [P2]
- Q33: *It was affected, the patient who does not have cars, the car is not available and there was a curfew and no taxi so how the patient can come? he cannot because of transportations*. [P8]
- Q34: *Many people hesitate to come not related to the safety of the clinic itself, but rather to many constraints including a curfew, people have less time to do many things during the day*. [P5]
- Q35: *Reducing crowding in the hospital, reducing exposure, reducing people who will catch covid or pass it, prevent what we call nosocomial (hospital spread) it was very helpful in terms of reducing covid risks but also freeing physicians and nurses to deal with covid related issues*. [P6]
- Q36: *It worked, it helped us to communicate with patients and help patients communicate with us. We were able to update them about their health and give them the right information about what to do with their sick or how to manage their chronic medical condition away from Clinic. We responded to their concerns and questions. We have them to refill their medications all these were done by these means of communication*. [P3]
- Q37: *Support all the staff in the hospital to improve telemedicine, give classes, to improve healthcare professional to improve telemedicine*. [P8]
- Q38: *Providing a good internet connection, is very important*. [P2]
- Q39: *The most important thing is that it is implemented well because if it is implemented well then people will love it and use it you don’t even have to convince them to use you just show it to them, and they will love it and use it*. [P7]
- Q40: *Establishing a law would make them feel better and would make them be more acceptance of it and it is all how you sell it*. [P1]
- Q41: *Form a group of interested people who would be interested in telemedicine with a representation of different institutions and different backgrounds and system design and software, engineers for sure, people who understand physical spaces and how that can be accommodated to enable telemedicine and physicians and clinicians as well*. [P6]
- Q42: *Having a distance conversation with my doctor without physically meeting him*. [C10]
- Q43: *All medical data about a person is stored in one place or a place where we can retrieve data. All the data from lab analysis results, to rays, to treatments, are stored in one place, or anyone can retrieve them from a specific place and then the doctor anywhere in the world in any health center. He sees the history and looks at all the treatments and he can prescribe treatments*. [C9]
- Q44: *You go to the appointment and find that the doctor is not in the hospital or took permission to leave or vacations and he did not say it before, so online much better support and it will make me stick to the appointment*. [C6]
- Q45: *When I am at work, I do not have to take permission, nor not going to work because I have to see the doctor no, I can finish the appointment in my workplace*. [C10]
- Q46: *I don’t believe the consultant does not know how to diagnosis, but in some cases, it cannot be done electronically like iris or pressure measurement he might have different equipment that what I have at home, they use more accurate equipment*. [C9]
- Q47: *Of course, it will be more comfortable, instead of you call them and arranging an appointment, and do not know when the appointment is or you go walk-in appointments that could annoy the patients' and might delay them by showing up between their appointments and someone misses his appointment, this way will be easier and more comfortable for people and people*. [C8]
- Q48: *Telemedicine is better in all stages, it is easier and more comfortable to let go of things like waiting in queue t and crowding, I mean, I can wait 3 or 4 hours to enter the doctor’s just for exactly 3 minutes follow up. but when it is online it is easier not only for me but also the doctor, I show him the reading results and then he decided if coming to the hospital is necessary*. [C6]
- Q49: *A face-to-face consultation, currently I see it as the best, but telemedicine certainly has goals, especially since we are Corona. We can benefit in the case that I have a great consultant outside Kuwait but for me, I prefer face-to-face*. [C9]
- Q50: *Of course, of course, it makes me keep the appointment because it will save me time and effort, and I will benefit*. [C7]
- Q51: *It will give me, easier access it will save money, it will save me time as well. And also, when I use telemedicine, it helps me to have entry to my data, an entry to my vitals on regular basis*. [C1]
- Q52: *I love to explore and discover and research the topic if it becomes official*. [C8]
- Q53: *I do not have the love of exploration; I wait for the results then decide*. [C5]
- Q54: *The examination is not accurate, other than face-to-face. It is possible that the doctor needs measurements. I can measure it with me, but the pressure device, for example, that I have is not like the one he has. It is a manual examination or when we need it, and this we lose by electronic medicine*. [C9]
- Q55: *There are no concerns... Except for the Internet it could disconnect*. [C3]
- Q56: *There must be a back-up and more printed, but in comparisons, you see it possible that there are many files that are lost by the government or, like when the warehouse was burnt, I see online, and it is easier because this is a record that I want to print*. [C6]
- Q57: *How will he manage to enter all the patients’ data, in diabetes we see the doctor less than the nurse or diabetes educator...even patients’ need to enter data if the blood sugar is not stable, but he does not write that in the logbook instead of in the application and see if the dosage was high or low, or maybe was it stress*. [C1]
- Q58: *It should not be complicated, because I do not have much experience with computers you know so I do not want complications I want it to be easy and when I open it, I want to have a direct way to reach the doctor with no complications*. [C3]
- Q59: *Maybe if someone enters my account and withdraws my data, I mean, for example, if there are blood tests inside and they pull the data and they know what I suffer from, and sometimes maybe the consultations are recorded with the doctor they can hear what I say to the doctor my fears or fears I have for the future, these are all information that I do not want anyone to know about*. [C6]
- Q60: It is possible that there are hackers and they hack the patients’ information, but at the same time, one should think that what do they want with my information *laughs* just to see my insulin levels? it is fine I will tell you my dose, maybe some people could get sensitive about it if they think the disease as a flaw but not me. [C8]
- Q61: *The fears are that there is an interference with the data, meaning in playing with the main data. This, if it happens in whether data is lost or played with data, will certainly affect the result, because it is data corruption or an error in data, so this is the confidentiality of storing information that no one can communicate the same to those who have an account in the bank The most important thing is that it is the bank’s security and its job again is the strength of the advisor himself that I will meet with him*. [C9]
- Q62: *With respect that there is no leakage between patients, the most important thing is honesty*. [C9]
- Q63: *The most important thing is speed and no lagging*. [C10]
- Q64: *The system should be compatible with the phone; some systems are not compatible with phones it gives you an error or cannot load the page because you are using it on your through phone or tablet*. [C1]
- Q65: *It should be simple, a report is sent via email for example if the system is hard to use, reminders for certain appointments and so and if we told them before, get notifications like ‘your report has been released’, your next appointment is tomorrow, accept the appointment so they don’t need to check on you by phone, you have the option to say yes or no, accept, reject*. [C1]
- Q66: *We could use the well-known social media networking, or we could design a special program for it, and the servers must be strong with minimum load errors and such, so prepare servers for this system*. [C8]
- Q67: *The language. I do not know English, so I ask my daughters to tell me what is written and translate for me, when the nurse does not know Arabic in the hospital I panic*. [C5]
- Q68: *To be honest, there is no effect on me, I see the doctor between three months, four months, sometimes times, five months*. [C10]
- Q69: *Yes, it did affect me, and I canceled my appointments during COVID-19 because I was afraid and did not check back because I did not want to go and get germs*. [C5]
- Q70: *Perhaps most of the things that happened to the dispensing of medicines, I did not need follow-ups with my doctor, but when I wanted to receive my medicine, the system was not well integrated, and they gave me the wrong medicine*. [C6]
- Q71: *It protected us diabetes patients the type where we did not have the protection, I mean why do I go to the hospital and catch epidemic from hospital or clinic, so this telemedicine has protected us it is like precautionary thing*. [C4]
- Q72: *The best thing was that it saved time, effort, and money. If appointments were online, it is better than going to the appointment in the hospital because online will reduce the spread of the virus*. [C7]
- Q73: *I think that is also a secondary way of doing things and we should look into it because we don’t know another pandemic may come so you have to be very much aware of that*. [C1]
- Q74: *It is possible that there are people who do not have much experience in technology, for example, they make lectures for people on how to use the program, educate the people because the technology as you said you can create it and use it however your like*. [C8]
- Q75: *Make interviews for different patients because they face problems different than mine, people are aware of other problems happening, from their perspective, and see many opinions the topic should be for public ... do questionnaire for all categories not just diabetes there are other diseases like immune deficiency let it be more general and make brochures or something for people to read if they have not done an interview so they are aware of it*. [C3]
- Q76: *First thing is honesty, do not to leak patient or patient information and to have fast service*. [C7]
- Q77: *The first recommendation is for the Ministry of Health to complete, the health issues, which is linking all the data of people in all centers. It is something very important, I collect all the information and put it in the system, and wherever I go to look at the data*. [C9]

##### Relative Advantages

In total, 6 participants mentioned how patients complain about the time they waste in the parking area and the waiting area (Q3) Likewise, 2 participants mentioned that telemedicine does not only save the patient's time but theirs as well (Q4). Further, 8 participants stated that telemedicine could improve clinical practices with patients (Q5).

##### Telemedicine Challenges

In total, 3 participants reported infrastructure as the main challenge (Q6). Along with infrastructure, 1 participant mentioned the importance of the implementation (Q7). The same participant mentioned that if we bring the patient to collect the papers there is no point in using telemedicine (Q8). In addition, 1 participant stated the importance of increasing technology awareness to increase the acceptance of telemedicine (Q9). Further, 4 participants mentioned technical issues and poor internet connection makes it difficult to rely on telemedicine (Q10). In total, 2 participants mentioned the importance of system integration (Q11).

##### Risks

In total, 1 participant mentioned that telemedicine is not risky if used along with physical visits (Q12). Of note, 6 participants mentioned that patients might misunderstand the given advice (Q13).

##### Trialability and Staff Acceptance

In total, 1 participant mentioned that her colleagues do not favor telemedicine for camera and trust reasons (Q14). While 7 participants reported their colleagues are interested in telemedicine (Q15).

#### Nonfunctional Requirements

##### Security and Confidentiality

In total, 1 participant mentioned that the security of the service is essential when it involves payment (Q16). In addition, 1 participant mentioned patients' records should remain confidential to their HCPs only (Q17).

##### User-Friendliness

Of note, 6 participants mentioned that user-friendly systems are what attract users of different ages (Q18).

##### Accessibility

In total, 1 participant mentioned more nonfunctional requirements attributes that should be supported (Q19).

##### Safe to Use

In total, 1 participant mentioned that telemedicine is safe to use under emergencies like COVID-19 but we need more data to determine whether telemedicine is safe to use under normal circumstances (Q20). In addition, 1 participant was uncertain of telemedicine use as it could result in a patient mix-up (Q21).

##### Older People Considerations

Of note, 4 participants mentioned that older people are advised to have a caregiver as they might have low levels of technology knowledge (Q22). In total, 1 participant mentioned that their workplace provides options for older people to choose from (Q23). In addition, 1 participant mentioned that older patients prefer to have visuals instead of audio so video consultations would be a better option as well as adjusting the website to be suitable for them (Q24).

##### Better System Recommendations

In total, 1 participant mentioned that telemedicine should be integrated with electronic health records (Q25). In addition, 1 participant mentioned that reducing clicks for systems is advisable (Q26). Further, 1 participant mentioned having visuals in the system to make it more friendly (Q27). Moreover, 1 participant recommends that the system should be easy (Q28). Furthermore, 1 participant mentioned there should be an alarm raised when the blood glucose is high or low (Q29). Notably, 2 participants suggest adding features for a patient with special needs and diabetes, such as sign language (Q30).

#### Telemedicine During Pandemics

##### Challenges During Lockdown

In total, 2 participants mentioned that one of the challenges they faced was finding the right timing to reach patients, sleep patterns, and not being honest with their answers (Q31). In total, 1 participant mentioned that the patient feared that they could not visit the doctor for a follow-up (Q32). Further, 2 participants mentioned that some patients faced transportation problems during the curfew (Q33). In addition, 1 participant mentioned that some patients had other obligations that they were committed to so they could not make it to the appointment due to time constraints (Q34).

##### Relative Advantages of Telemedicine During the Pandemic

In total, 10 participants agreed that telemedicine during the pandemic has its advantages for patients’ safety (Q35). In total, 1 participant mentioned that they were able to reach the patients and provide them with treatment and updates (Q36).

#### Recommendations

In total, 3 participants suggested educating the staff and patients through lectures and workshops (Q37). Further, 3 participants mentioned establishing good service and a strong internet connection (Q38). In total, 1 participant focused on the importance of implementation because if it is not well implemented then no one will use it (Q39).

In total, 1 participant mentioned that there should be laws and regulations when using telemedicine (Q40). Further, 1 participant mentioned forming a group of individuals who are interested in telemedicine (Q41).

### Perspectives of Patients With Diabetes

#### Background

All 10 patients were aware of the definition of telemedicine; 1 participant stated (Q42). One of our participants was a computer programmer who defined telemedicine from his perspective (Q43).

#### Challenges and Opportunities

##### Relative Advantages

In total, 1 participant mentioned that it is easier to cancel an appointment on the internet rather than going to the hospital and waiting for the appointment to receive the cancelation (Q44). Further, 2 participants mentioned an advantage of telemedicine by not having to take permission to leave work to attend an appointment, it can be done in the workplace (Q45).

##### Telemedicine Challenges

In total, 3 participants mentioned that diagnosing medical complaints from a patient with diabetes can be a challenge (Q46).

##### Patients With Diabetes Who Experienced Telemedicine

In total, 4 participants have experience with telemedicine. All 4 participants agreed that they had a comfortable feeling communicating with their health provider on the internet (Q47). Further, 2 participants prefer telemedicine appointments rather than face-to-face ones (Q48). Furthermore, 2 participants preferred face-to-face consultation but do favor using telemedicine in some situations (Q49).

##### Observability

All 10 participants agree that web-based appointments would encourage them to keep up with the appointment (Q50). All 10 participants agreed that telemedicine will save time and money (Q51). Of note, 7 early adopter participants mentioned that they would like to explore telemedicine by themselves (Q52). Further, 2 laggards mentioned that they would rather observe the results of telemedicine rather than directly adopt it (Q53).

##### Concerns

In total, 3 participants mentioned that their concern is that there might be a misdiagnosis or misunderstanding (Q54). In total, 1 participant said that internet connection could be a concern (Q55). In addition, 1 participant mentioned that there should be backup data that he could print himself (Q56). In total, 1 participant was concerned about data entry and how the doctor will enter each patient’s data with minimum visits (Q57).

#### Nonfunctional Requirements

##### User-Friendliness

In total, 7 participants noted that telemedicine applications must be easy to use (Q58).

##### Security and Data Integrity

Of note, 4 participants mentioned they care about security and confidentiality (Q59). Further, 2 participants mentioned that security is not a concern to use telemedicine (Q60). In total, 1 participant mentioned the importance of how to preserve the data in telemedicine (Q61). The same participant mentioned that honesty is also a factor that telemedicine should include (Q62).

##### Performance and Availability

In total, 5 participants mentioned that telemedicine should be fast performance and available most of the time (Q63).

##### Compatible

In total, 2 participants mentioned that telemedicine should be compatible with different devices (Q64).

##### System Features

In total, 1 participant mentioned that sending appointment reminders, and notifications would be appreciated (Q65). In addition, 1 participant mentioned that servers for the system should be strong with minimum errors (Q66). Further, 3 participants mentioned that telemedicine should support the mother language (Q67).

#### Telemedicine During Pandemics

##### Lockdown Appointments Challenges

In total, 2 participants mentioned that the lockdown did not have any effect on their appointments (Q68). Of note, 7 participants mentioned that they were worried about catching COVID-19 so they canceled the appointments (Q69). Further, 1 participant mentioned that he did not have a problem with appointments as much as problems with receiving the medicine (Q70).

##### Relative Advantages for Telemedicine During a Pandemic

All 10 participants agreed that telemedicine has many advantages during the pandemic—in our case COVID-19 (Q71). Of note, 5 participants mentioned that telemedicine will reduce virus spread as well as save time and effort (Q72). In total, 1 participant mentioned that telemedicine is secondary, and we need to have it ready if another pandemic occurs (Q73).

#### Recommendations

Of note, 4 participants mentioned that people need to be educated about telemedicine and how to use it through lectures (Q74). In total, 1 participant reported that researchers should do more interviews and surveys to have a complete understanding of patients' perspectives (Q75). In addition, 1 participant mentioned that honesty is a priority (Q76). Moreover, 1 participant suggested that the Ministry of Health should link the health data across health centers (Q77).

## Discussion

### Principal Findings

The results of this study showed that the majority of the participants, including HCPs and patients with diabetes, are aware of and eager to adopt telemedicine technology. This is the first study that explores the challenges and opportunities of telemedicine adoption for diabetes care and management in Kuwait. Patients with diabetes require frequent monitoring and appointments, which can be challenging to manage in person. Telemedicine provides a solution for HCPs to deliver medical care remotely through video calls on mobile or computer devices.

Previous studies have used telemedicine videoconferencing and in-person consultations for patients with diabetes to evaluate the impact on hemoglobin A_1c_ levels and to help patients improve their management through lifestyle changes [35]; although a small decrease was noted in both groups, patients who used telemedicine showed more satisfaction [36,37].

Not all our participants with diabetes have experienced telemedicine, however, they were more enthused to attempt it. Notable patients’ responses to the challenges of adopting telemedicine are that the physicians might misdiagnose their health condition and may not provide accurate information. However, the fear of misdiagnosis is also present in patients who hesitate to seek physical medical care in the first place [10,38].

HCP participants in this study have illustrated the importance of building trust between HCPs and patients for telemedicine to succeed as a reliable method for delivering health care [39]. According to similar studies, the researchers found that patients would be more inclined to use telemedicine if they could communicate with the same HCP they were already comfortable with in person, thus helping them accept telemedicine [40-42].

A crucial key to continuously using telemedicine in health care systems is to pay attention to the nonfunctional requirements mainly focusing on ease of use and user-friendliness. The participants in this study were from varying age groups and pointed out that to use the system needs to be easy and friendly to use; similar results were found in a recent study that used telemedicine for patients and caregivers with head and neck conditions who reported that they were satisfied with using it because of how easy it was to use [43].

Security requirements were controversial; patients were either concerned that physicians may misuse their data or patients would not mind third parties viewing personal data. Similar studies recommend producing a well-planned implementation structure for the adoption of telemedicine according to the regulations for protection [44,45].

COVID-19 raised the need of adopting telemedicine across the globe to reduce the spread of the virus among patients, especially with patients with low immunity as well as to stay in communication with patients during lockdowns. Some of our participants have been introduced to web-based consultations through phone calls and social media communication applications resulting in feedback that it was satisfying in terms of time-saving. A recent study explained that telemedicine maintains unnecessary social distances yet succeeds at disaster management [46,47]. HCPs report that one challenge facing the adoption of telemedicine is the staff’s insufficient knowledge of technology, and reported that it can be time-consuming for staff to change workflows [48,49].

Surprisingly, older patients in our study gave positive and supportive feedback regarding the use of telemedicine, not to mention that one of the HCPs shared that older patients are good with using technology. Age is not a factor to limit telemedicine to a certain group age as similar studies found that older patients are using technology [50]. HCPs have mentioned that patients with disability and diabetes require more attention and need to focus on adding design and functional features [51].

### Recommendations

To enhance the adoption and usage of telemedicine for diabetes care and management, participants in this study have provided suggestions for relevant stakeholders. IT staff can work together to provide educational sessions, training workshops, and hands-on experience for both HCPs and patients with diabetes to ensure they are ready for the new system and can provide feedback for continuous improvement [52]. Distributing surveys after telemedicine implementation can help to better understand how users feel about it and how it has affected their treatment, it can be useful to measure the level of population acceptance of telemedicine adoption.

Policy makers are required to generate new regulation laws and ethical concerns to help minimize the risks of telemedicine consumption and help protect HCPs and patients' medical rights. Another recommendation to minimize risks and concerns for the users is to focus on a good implementation strategy. Because telemedicine needs internet to work, it is vital to provide strong internet connection in hospitals, this will ensure better performance, as well as ensure a high scalability system.

Because telemedicine is a useful tool to deliver medical care to remote locations, there are opportunities to expand the reach of care services to patients with diabetes across the globe and provide the services they require conveniently. Additionally, developers of telemedicine platforms should consider borrowing similar design concepts from social media platforms due to many people being familiar with using such platforms for communication purposes, which in turn can ensure ease of use of telemedicine [53].

### Study Strengths and Limitations

Like other studies, this research has certain limitations. The participants were limited to those over 21 years old, and further research is needed to understand the perspectives of adolescent patients on telemedicine adoption. Although the patients with diabetes in this study were eager to adopt telemedicine and recruited through social media, more interviews with a diverse range of socioeconomic backgrounds would be necessary to understand those who do not support telemedicine. The COVID-19 pandemic has limited the outreach to this study’s population and relied on the use of social media and web-based communication platforms to recruit participants. This study aimed to gain in-depth knowledge on the topic, not to generalize the results, as there is limited evidence available. While the findings may relate to similar cultural contexts of neighboring nations, caution should be exercised before assuming applicability. Future research could gather data from a larger and more diverse group of patients with diabetes and HCPs in various settings (ie, health system organization, population characteristics, and cultural context).

### Conclusions

The findings of this study provide important insights into the challenges and opportunities of adopting telemedicine for diabetes care and management in Kuwait, including during the times of health emergencies such as the COVID-19 pandemic. This qualitative and exploratory study sheds light on the perspectives of both patients with diabetes and HCPs regarding the adoption and use of telemedicine in Kuwait. Participants were generally familiar with and interested in using telemedicine and noted its benefits, such as saving time and increasing patient safety. However, they also highlighted the importance of ensuring a secure and user-friendly system, as well as providing education and training for HCPs and patients with diabetes. Policy makers and HCPs should consider these findings as they work to improve the adoption and use of telemedicine for diabetes care in Kuwait.

## Appendix

Multimedia Appendix 1 Health care professionals' interview questions.

Multimedia Appendix 2 Patients with diabetes interview questions.

## Abbreviations

HCP: health care provider
NCD: noncommunicable disease
