# Supplementary material for: Exploring the Challenges and Opportunities of Adopting and Using Telemedicine for Diabetes Care and Management: Qualitative Semistructured Interview Study Among Health Care Providers and Patients With Diabetes
Source: JMIR Hum Factors. 2023 Sep 7;10:e46324. doi: 10.2196/46324 (PMC10514770; doi:10.2196/46324)
Supplement: Multimedia Appendix 1 [file humanfactors_v10i1e46324_app1.docx]

**Healthcare Professional Interview Guide (21 Years and Older)**

# [Read consent form. Only proceed upon agreement from the participant.]

# Demographics

1. Gender.
2. How old are you?
3. What is your role/specialty?
4. Years of professional experience?
5. Where do you work? What type of organization do you work for?
6. Approximately how many patients does your organization see? OR how many employees are in your department/organization?

# Background

1. What health IT solutions are available in your workplace? What do you use? [EHRs, RIS/PACS, LIS, … etc.] **observability**
2. Do you provide any services to your patients with diabetes remotely?
   - *Examples WhatsApp, email, …*
3. Are you familiar with the term telemedicine? [if not known, provide definition]
4. Do you use telemedicine? **Compatibility**
5. What do you think are the main benefits of telemedicine? **Relative advantage**

# Challenges and Opportunities

1. Do you see value in adopting telemedicine in Kuwait for diabetes and other related chronic diseases? Why/why not? **Relative advantage**
2. How do you think this technology would be useful for you? **Compatibility** (Tasneem, Kim, Bagheri, & Lebret, 2019)
3. How can telemedicine improve your clinical practice? Can you provide us with examples? (Yaghobian, Ohannessian, Mathieu-Fritz, & Moulin, 2019) **Compatibility**
4. How do you expect the performance of telemedicine if applied in your workplace compared to standard care service? Please provide answers relating to the quality and availability of the care service. **Relative advantage**
5. Will your colleagues be interested in this modern technology? Why/why not? **Trialability**
6. Can you comment on the readiness of patients for telemedicine in Kuwait? What do you expect their attitude to be towards using this technology? **Compatibility/ Complexity**
7. What are the potential reasons for patients to resist using telemedicine? **Relative advantage**
8. What do you perceive to be the risks associated with using telemedicine for diabetes care and management? **Compatibility/ observability**
9. What are the top three challenges/barriers you see for adopting and using telemedicine for diabetes care and management in your practice? In Kuwait?
   - *Probes: Technical support, infrastructure, training, cost, complexity, leadership support, and sponsorship … etc.*

# Non-functional Requirements

1. From your point of view, what requirements are important for you to use telemedicine in your practice?
   - *Probes: security, availability, performance, Interoperability, usability, … etc.*
   - *Probes for security: ask for password frequently/ logout after certain minutes passed*
2. Would you consider telemedicine safe to use? Why/why not?
3. Are there particular considerations for telemedicine and its use for elderly patients who have diabetes or special needs?
   - *Probes: deaf, blind, the ability to zoom-in, voice built-in, sign language*
4. What are your recommendations for better system design?
   - *Probes: reminder messages, easy navigation*

Telemedicine during pandemic

1. How did the lockdown affect your appointments with patients?
   - Patients requesting online alternatives
2. Was there any technology used to communicate with the patients during lockdown?
   - If so, what were the challenges?
3. What are your opinions regarding the benefits of telemedicine during pandemic such as COVID-19?

# Recommendations

1. What are some recommendations that you would give to promote telehealth adoption and use for diabetes care and management in Kuwait?
   - *Can you share the top 3 recommendations for improving telemedicine adoption for diabetes care and management in Kuwait?*
   - *Policy and strategy recommendation.*
   - *Probes: funding, enough devices staff with experience*

# Others

1. Final question: Is there anything else you would like to add?

***[Thank you for participating in this interview. Once I complete the transcription of the interview and conduct a preliminary analysis, I may contact you for clarification purposes if that is OK. We truly appreciate your time.]***

***If the participant agrees, take their preferred contact information.***
