# Supplementary material for: Exploring the Challenges and Opportunities of Adopting and Using Telemedicine for Diabetes Care and Management: Qualitative Semistructured Interview Study Among Health Care Providers and Patients With Diabetes
Source: JMIR Hum Factors. 2023 Sep 7;10:e46324. doi: 10.2196/46324 (PMC10514770; doi:10.2196/46324)
Supplement: Multimedia Appendix 2 [file humanfactors_v10i1e46324_app2.docx]

**Patient Interview Guide (21 Years and Older)**

[Read consent form. Only proceed upon agreement from the participant.]

# Demographics

1. Gender
2. How old are you?
3. Your current status [student/employee/retired]?
4. Highest degree obtained
5. Where do you work/study?
6. What type of diabetes do you have?
7. How long have you been living with diabetes?
8. How controlled is your diabetes?
9. How often do you see your clinician to follow-up on your diabetes?

# Background

1. Do you own a smartphone? Are you subscribed to the Internet?
2. Do you use any apps to help you manage your diabetes? Which ones? Can you show me?
3. Are you familiar with the term telemedicine? What does it mean to you? [provide the definition if not]
4. Do you use or have you used telemedicine? Have you ever had a telehealth consultation?
   - If yes:
     1. How did you feel about the consultation?
     2. How did it compare with a face-to-face consultation?
     3. Were you comfortable with the consultation?
     4. Were you confident the consultation was done just as well as if you saw the doctor in person?
     5. Did you understand the advice you were given?
     6. Will you have further consultations this way?
5. Do you see value in adopting and using telemedicine in Kuwait for your condition? Why/why not?

# Challenges and Opportunities

1. How would you feel about talking through video with your specialist? (Tasneem, Kim, Bagheri, & Lebret, 2019) **Trialability**
2. How will the telemedicine visit impact your health care? (Tasneem et al., 2019) **Observability**
3. How do you think this technology would be useful for you? **Compatibility** (Tasneem, Kim, Bagheri, & Lebret, 2019)
4. Would you feel that a telemedicine visit will save you time, money, etc.? If so, how? (Tasneem et al., 2019) **Relative Advantage**
5. Would it make it more likely for you to keep an appointment with your doctor? Why/why not? (Tasneem et al., 2019) **Observability**
6. Would you prefer to explore the telemedicine system yourself or would you rather observe others first? **Trialability**
7. **What are your concerns when using telemedicine? Complexity**

# Non-functional Requirements

1. From your point of view, what requirements are important for you to use telemedicine to manage your condition?

*Probes: security, availability, performance, usability, interoperability … etc.*

1. What are some concerns you may have about your privacy or confidentiality about using this system to meet with your doctor? (Tasneem et al., 2019)
2. What are your top 3 suggestions to make telemedicine easy for patients to use?

Telemedicine During pandemic

1. How did the lockdown affect your appointments with the doctor?
2. What are your opinions regarding the benefits of telemedicine during pandemic such as COVID-19?

# Recommendations

1. Can you share the top 3 recommendations you have for increasing the adoption of telemedicine in Kuwait for diabetes care and management?

# Others

1. Final question: Is there anything else you would like to add?

***[Thank you for participating in this interview. Once I complete the transcription of the interview and conduct a preliminary analysis, I may contact you for clarification purposes if that is OK. We truly appreciate your time.]***

***If the participant agrees, take their preferred contact information.***
